# Comparative Evaluation of the Effectiveness of Rotary and Reciprocating Single File Systems on Postoperative Pain After Biomechanical Preparation in Patients With Chronic Apical Periodontitis: A Randomized Clinical Trial

**DOI:** 10.7759/cureus.72411

**Published:** 2024-10-26

**Authors:** Pooja K P, Jayasree S, Elizabeth P James, Ajeesh K, Shareefa B, Maneesha K, Fathima Jauhara

**Affiliations:** 1 Department of Conservative Dentistry and Endodontics, Government Dental College, Kozhikode, IND

**Keywords:** biomechanical preparation, chronic apical periodontitis, postoperative pain, reciprocating single file system, rotary single file system, single rooted tooth

## Abstract

Background and objective

Postoperative pain after biomechanical preparation of root canals can be equally distressing to the patient as well as the dentist and can mold the patient's attitude and trust for future dental procedures. This randomized clinical trial aimed to evaluate the effectiveness of rotary and reciprocating single-file systems on postoperative pain after biomechanical preparation in patients with chronic apical periodontitis.

Materials and methods

In this randomized clinical trial (CTRI Reg No-CTRI/2021/04/032841), 94 patients with chronic apical periodontitis in single straight-rooted teeth were divided equally into two groups, according to a standardized protocol. Biomechanical preparation of the root canal was done by a single operator using the Hyflex Electrical Discharge Machining (EDM) rotary file system (Coltene, Altstätten, Switzerland) in group 1 and Reciproc Blue (VDW Dental, Munich, Germany) file system in group 2, respectively. Standardized instrumentation and irrigation techniques were followed in both groups. Participants (blinded to the instrumentation technique) rated their pain intensity at 24 hours, 48 hours, and 72 hours following the root canal instrumentation appointment using the Visual Analog Scale (VAS) and Numerical Pain Rating Scale (NPRS).

Statistical analysis

The number and time of analgesic intake were also recorded. The data was analyzed using repeated ANOVA, post hoc test, Student T-test, and independent T-test at a 5% level of significance with 80% power of the study.

Results and discussion

Both the file systems showed low mean postoperative pain intensity at all three time-points assessed. The mean pain value was found to be greater in the Hyflex EDM group at 24 hours, but less at 48 hours and 72 hours when compared to the Reciproc Blue group. However, this difference in pain values was not statistically significant at any of the assessed time points (p>0.05). No significant difference in pain was found based on gender and analgesic intake between the two groups (p>0.05).

A low incidence of postoperative pain detected in this study can be attributed to the advanced endodontic devices and methods used during routine endodontic procedures, which provide more appropriate chemo-mechanical disinfection.

Conclusion

There was no statistically significant difference in postoperative pain between the rotary single file system and reciprocating single file systems at all the assessed time intervals. Hence, this study concluded that the instrumentation kinematics (single-file reciprocating or single-file rotary) had no impact on the intensity of postoperative pain after biomechanical preparation, and no file system is superior to the other in terms of postoperative pain, and hence, both file systems can be used clinically with equal efficiency when considering postoperative patient discomfort

## Introduction

Postoperative pain after biomechanical preparation during root canal treatment can be equally distressing to the patient and dentist, similar to the pain after the endodontic treatment.

Extrusion of infected debris into the periapical tissue during endodontic treatment is a well-established factor behind postoperative pain [1].

Even though the newer technologies in file systems and their kinematics have claimed to reduce apical debris extrusion to a large extent, it is an inevitable process during root canal preparation [2]. This scenario also warrants the requirement of a highly sensitive periapical environment to assess the outcome of such a small amount of debris extruded. Hence teeth with chronic apical periodontitis were considered ideal candidates to assess the same due to the fragile mechanism of "local adaptation syndrome" seen in their periapical region [3].

The present study performed root canal preparation with Hyflex Electrical Discharge Machining (EDM) (Coltene, Altstätten, Switzerland) and Reciproc Blue (VDW Dental, Munich, Germany) single file systems in the rotary and reciprocating group, respectively, both of which their manufacturers claim to have better physical and metallurgical properties than their predecessors [4,5].

HyFlex EDM (HEDM) controlled memory alloy files were made using electrical discharge machining technology [6]. Hyflex EDM file system consists of a set of three files, that is, an orifice opener (25/0.12), glide path file (10/0.05), and shaping file (25/0.08).

Reciproc Blue was claimed to be more flexible, causing reduced apical debris extrusion than its predecessor. They are available in three different sizes and tapers, that is, R 25 (25/.08), R 40 (40/.06), and R 50 (50/0.05) [7].

This study aimed to evaluate the occurrence and intensity of pain in patients with chronic asymptomatic apical periodontitis after biomechanical preparation of root canals with rotary and reciprocating single-file systems. The findings of this study will help the clinician to choose the best file system available to maintain the patient in a pain-free state and reduce patient anxiety and stress in the subsequent appointments, making it a pleasant experience for both the patient and the dentist and alleviating the patients "fear of unknown" in the future dental procedures.

## Materials and methods

This parallel grouped double-blinded randomized clinical trial was done in the Conservative Dentistry and Endodontics Department of Government Dental College, Kozhikode. The study was approved by the Institutional Ethics Committee of Government Dental College Kozhikode (register no: ECR/673/Inst/KL/2014/RR-20) with IEC no:216/2020/DCC, dated 21/12/2020 and was registered with the Clinical Trials Registry-India (ICMR-NIMS) with CTRI Reg No-CTRI/2021/04/032841.

Sample size calculation

To detect a clinically relevant difference of 0.4 at a 5% level of significance with 80% power, the required sample size was arrived at 47 samples in each group. The sample size was calculated based on a previous study by Alomari et al. [8], comparing the effectiveness of two file systems with different motion kinetics in determining postoperative pain after root canal treatment.

Participant selection and allocation

**Figure 1:**
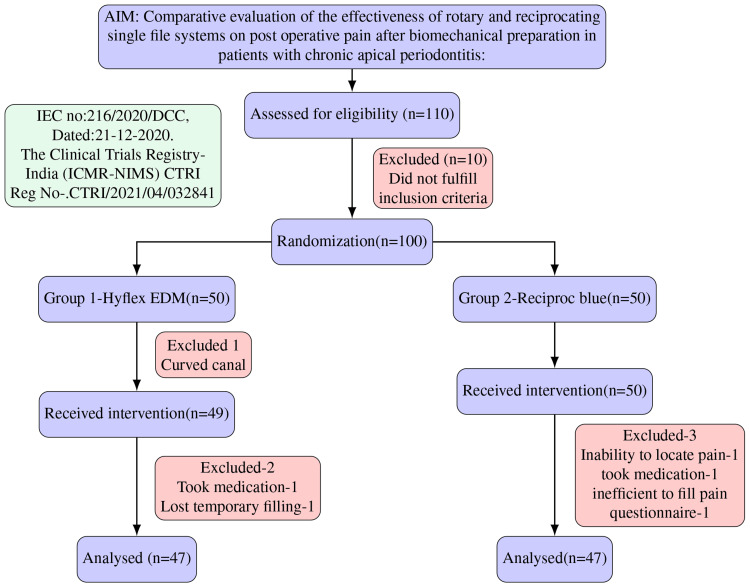
: CONSORT flow diagram of the clinical trial CONSORT - Consolidated Standards for Reporting Trials; n - sample size (n=47 corresponds to 100% sample size)

The protocol followed the recommendations of the Consolidated Standards for Reporting Trials (CONSORT) statement (Figure 1).

Study population

The study population consisted of patients within the age group of 20 to 40 years, reporting to the Department of Conservative Dentistry and Endodontics, Government Dental College, Kozhikode, Kerala.

Randomization and group allocation

After obtaining written consent before treatment, the tooth was allotted to one of the instrumentation groups based on a sealed envelope method by a dental assistant blinded to the study. Block randomization, with a block size of four, was done. The selected participant was excluded from the study when the tooth did not fulfill the inclusion criteria during any procedure stage. A new participant was then included in the study to compensate for the lost one.

Inclusion criteria

The study population consisted of patients from both genders aged 20 to 40 years with a diagnosis of chronic apical periodontitis with periapical lesion size less than 3mm in single-rooted teeth with a single straight canal. 

Exclusion criteria

Teeth without good apical constriction, periodontal pathosis, and weakened periodontium, complex root canal anatomy, presence of sinus tracts, retreatment cases, patients on medication for chronic pain, adjacent teeth requiring root canal therapy, medically compromised patients, teeth with long roots and wide canals, infectious diseases and history of taking drugs one week before treatment were excluded from the study.

Methodology

During the procedure, the tooth was anesthetized using 2% of lignocaine with 1:200000 epinephrine (Health Biotech, Solan, India). Occlusal reduction was done in all cases. Occlusal contact on the functional and non-functional cusp, as well as marginal ridges, were reduced by 1mm using a wheel diamond bur in a high-speed handpiece with copious coolant. The presence or absence of contact was confirmed using articulating paper (Jaypee) where occlusal surfaces were dried and articulating paper was held between the teeth with the mandible guided to a centric position. The tooth was then isolated with a rubber dam (Coltene, Altstäatten, Switzerland).

Access cavities were prepared using round diamond burs in a high-speed handpiece in both study groups. The tooth was allocated into any of the groups if a size 15 k file fit passively into the canal. A glide path was prepared in all the cases. The lengths of the canals were determined using an electronic apex locator (Root ZX Mini apex locator, J. Morita, Tokyo, Japan) and then confirmed radiographically with a radiovisuography image (RVG) using the bisecting angle technique. In case of an irregular root outline or suspected root curvature, mesial and distal shifts were taken.

**Figure 2:**
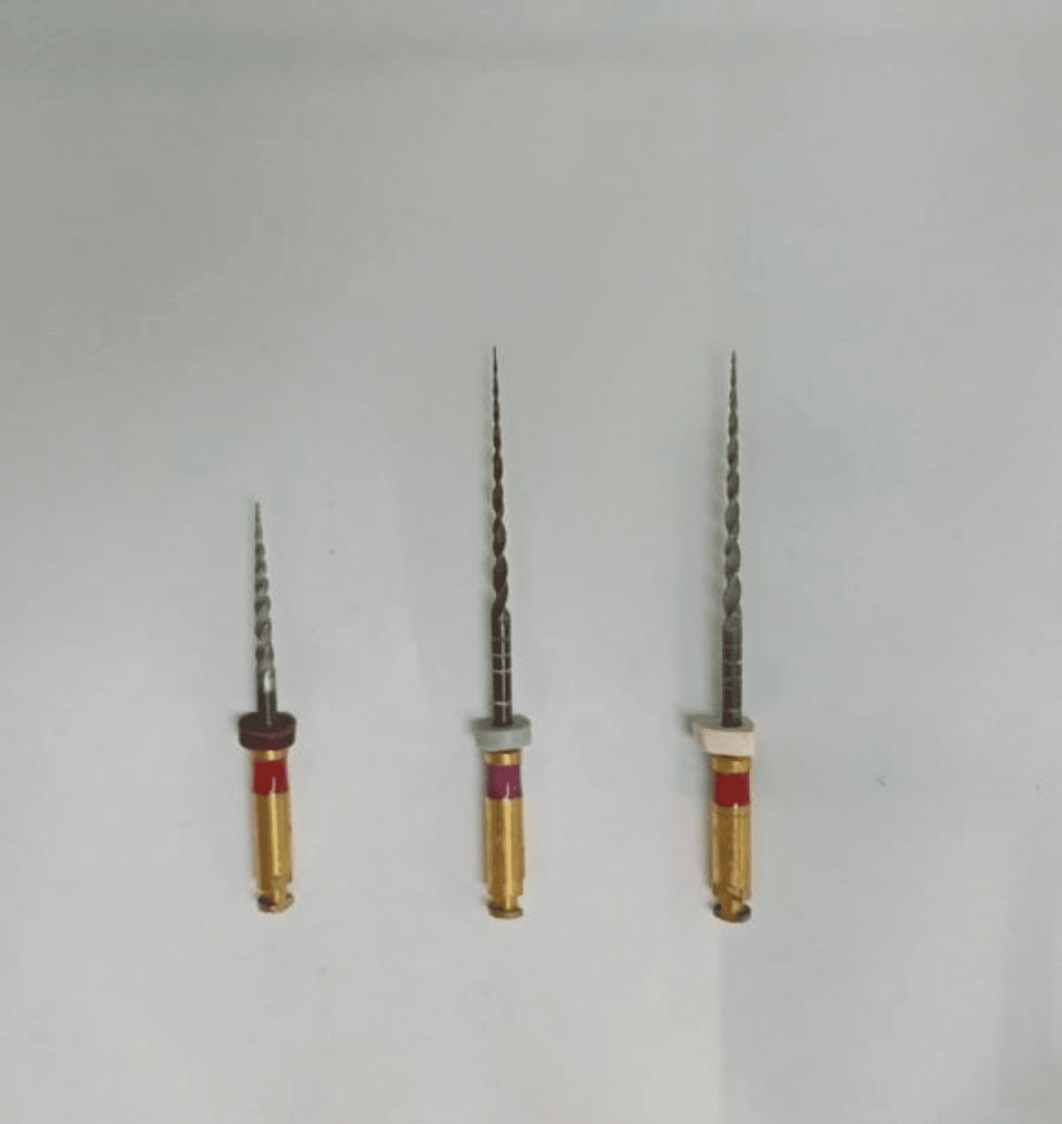
: HyFlex EDM file system - pack of 25/0.12 orifice opener, 10/.05 glidepath file, and 25/~ HyFlex EDM OneFile EDM - Electrical Discharge Machining

**Figure 3:**
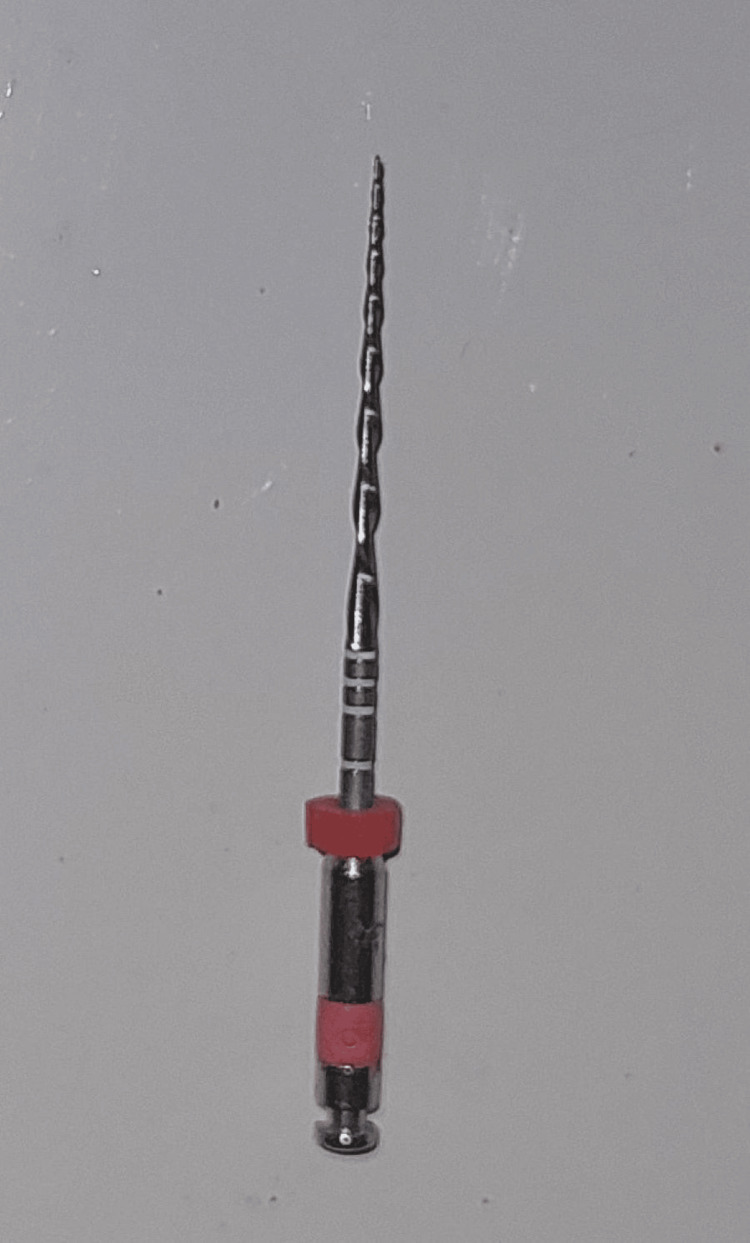
: Reciproc Blue file system - 25/.08 R25 single file system

**Figure 4:**
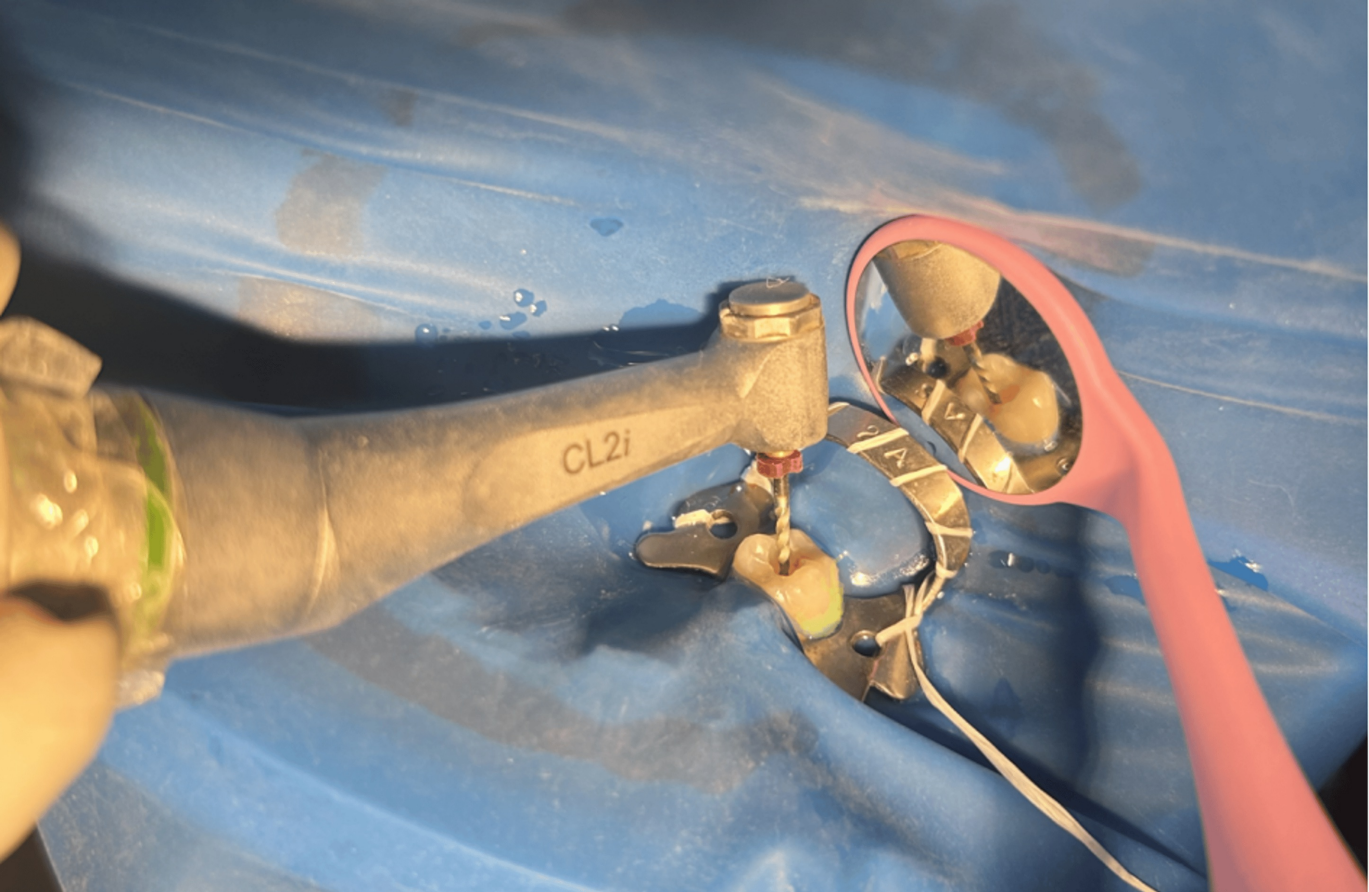
Biomechanical root canal preparation in group 1 - HyFlex EDM orifice opener (25/0.12) at 400 rpm speed and 2.5 Ncm torque Group 1 - rotary single file system; EDM - Electrical Discharge Machining

**Figure 5:**
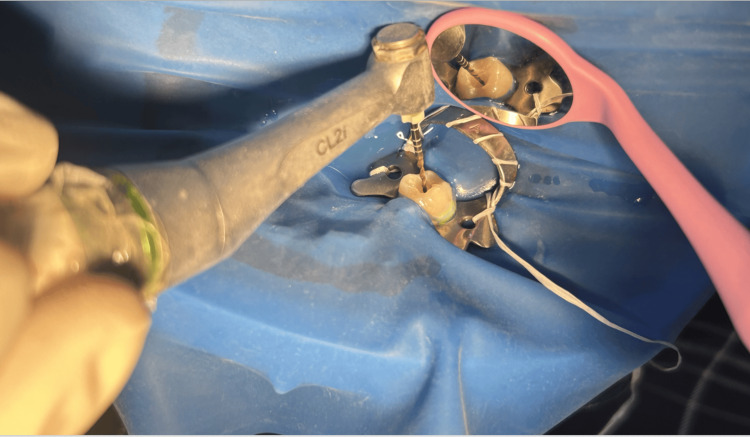
Glide path preparation with 10/05 HyFlex EDM Glidepath file at 300 rpm speed and 1.8 Ncm EDM - Electrical Discharge Machining

**Figure 6:**
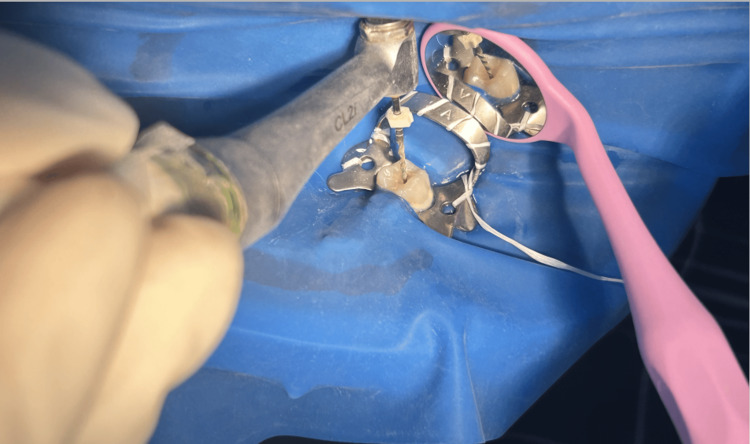
Root canal preparation with HyFlex EDM 25/0.08 single file at continuous rotation Gentle apical strokes and pecking movements in accordance with the recommendations of the manufacturer (continuous rotation at 400 rpm speed and 2.5-Ncm torque value) were used to prepare the canals with file 25/0.08 EDM - Electrical Discharge Machining

**Figure 7:**
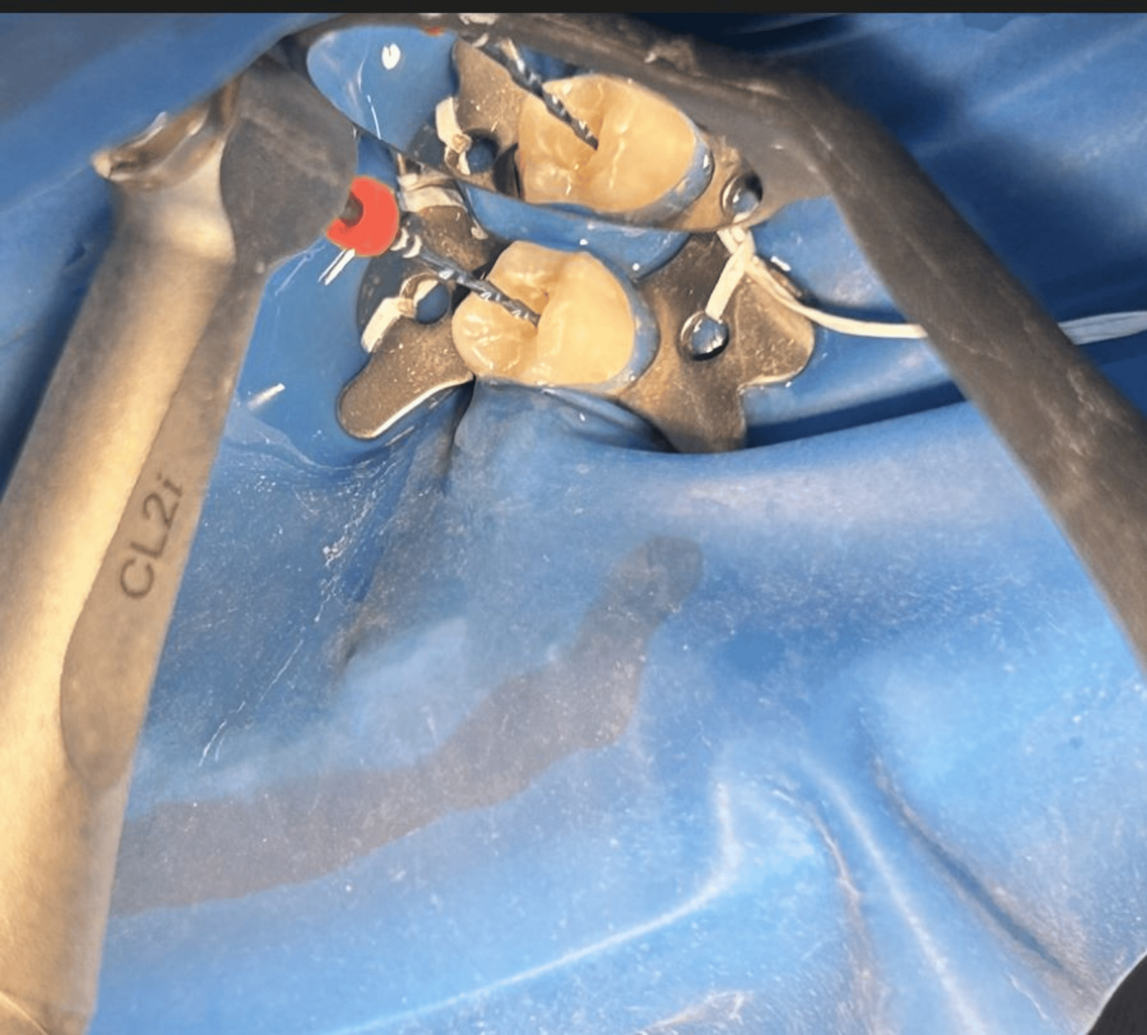
Biomechanical preparation of root canal in group 2 - Reciproc Blue R25 file (25/.08) was used to complete the root canal shaping Group 2 - reciprocating single file system Three in and out motions were applied with stroke lengths not exceeding 3 mm in the cervical, middle and apical thirds until attaining the established working length

Root canal preparation was done using Hyflex EDM rotary single file system (Figure 2) in group 1 and Reciproc Blue reciprocating single file system (Figure 3) in group 2, respectively. A glide path was prepared in all the cases. The lengths of the canals were determined both using an electronic apex locator (Root ZX Mini, J. Morita, Tokyo, Japan) and radiographically. The instrumentation sequence in both groups followed the procedure recommended by the manufacturer. CanalPro CL2i endomotor (Coltene Altstäatten, Switzerland) was used for biomechanical preparation in both file groups at manufacturer-recommended speed and torque (Figures 4-7)

During preparation, the canal was flushed with 5 mL of 5.25% NaOCl. After preparation, the final irrigation of each root canal included flushing with 2.5 mL of 17% EDTA, 5 mL of saline, and 2.5 mL of 5.25% NaOCl, respectively. Finally, NaOCl was flushed out with 10 ml of saline, and the canal was dried with sterile paper points. Calcium hydroxide intracanal medicament was given in all cases, and a temporary filling with intermediate restorative material (Dentsply Sirona) was given. Patients were prescribed an optional ibuprofen analgesic of 400mg and were asked to record the information if they took the medication.

Postoperative pain was assessed using the Visual Analogue Scale (VAS) and Numerical Pain Rating Scale (NPRS) at 24 hours, 48 hours, and 72 hours.

Visual Analogue Scale (VAS) pain score indicates the following; a score of 0-4mm indicating no pain, 4.1mm -4.5cm indicating mild pain, 4.6-7.0cm indicating moderate pain, and 7.1-10cm indicating severe pain.

Numerical Pain Rating Scale (NPRS) pain scores indicate the following; a score of 0 indicating no pain, 1-3 indicating mild pain, 4-6 indicating moderate pain, and 7-10 indicating severe pain.

After giving detailed instructions on how to fill the pain chart, all patients who met the final inclusion criteria were given a pain chart to be filled from home to record the occurrence and intensity of pain at 24 hours, 48 hours, and 72 hours post-treatment. Patients were reminded over the phone to fill out the pain charts at the required time intervals (24 hours and 48 hours). Patients were asked to submit the pain charts (Visual Analog Scale and Numerical Pain Rating Scale) after three days when reporting for the next appointment. The score of the Numerical Pain Rating Scale (NPRS) was reconfirmed by calling the patient over the phone at 24 hours and 48 hours by an evaluator blinded to the study. Patients were made to fill the NPRS and VAS at 72 hours directly before the evaluator blinded to the study. These helped the evaluator assess the patient's skill and understanding of filling the pain charts.

Patients reporting with dislodged or lost temporary restoration, analgesic intake, those unable to locate the source of pain, and those patients who showed inefficiency in filling the pain charts were excluded from the study. The evaluator kept the data from pain charts obtained from the patients blinded to the study group. Once the sample size was reached, these data were directly given to the statistician, who was also blinded about the allocated group.

Statistical analysis

The collected data were subjected to statistical analysis using SPSS version 25 software at p<0.05 (IBM Inc., Armonk, New York). Intra-group comparisons of pain at different time points were made using repeated measure ANOVA and comparison between each pair using post-hoc test. Intergroup comparison of pain at 24 hours, 48 hours, and 72 hours was done using the Student t-test, and comparison of the difference in means of pain between two groups using an independent t-test.

## Results

Patients were in the age group of 20-40 years, with a mean age of 27.8 years in group 1 and 28.7 years in group 2. The male-to-female participant ratio was 45:55 in group 1 and 40:60 in group 2. The tooth analyzed in group 1 was 35 mandibular second premolars, six upper canines, and six mandibular first premolars. Group 2 consisted of 36 mandibular second premolars, four upper canines, and seven mandibular first premolars. The average mean pain measurement value was 1.09 in group 1 and 0.92 in group 2 at 24 hours, 0.4 in group 1 and 0.52 in group 2 at 48 hours, and 0.17 in group 1 and 0.24 in group 2 at 72 hours. The pain intensity was slightly higher in the rotary group at 24 hours but showed a slight decrease in the reciprocating group at 48 hours and 72 hours (Table 1). However, the two groups had no statistically significant difference in mean pain values at any of the three time-points assessed (p>0.05; Table 2). The reduction in mean pain value from 24 hours to 48 hours and 48 hours to 72 and 24 hours to 72 hours were statistically significant in both group 1 (Table 3) and group 2 (Table 4; p<0.05).

**Table 1:** Inter group comparison of pain value between group 1 (rotary single file) and group 2 (reciprocating single file system) Group 1 - rotary single file system; group 2 - reciprocating single file system; VAS - Visual Analogue Scale; NPRS - Numerical Pain Rating Scale At 24 hours the mean value of pain was found to be slightly high in the rotary group when analyzed using both VAS and NPRS. At 48 hours and 72 hours, the mean value of pain was found to be slightly lesser in the rotary group than Reciproc group when measured using both VAS and NPRS.

	Group	N	Mean	Std. Deviation
VAS 24 hours	Group 1	47	1.1163	1.22279
	Group 2	47	0.9837	0.98051
NPRS 24 hours	Group 1	47	1.0698	1.00937
	Group 2	47	0.8605	0.8042
VAS 48 hours	Group 1	47	0.3442	0.63d931
	Group 2	47	0.4833	0.67351
NPRS 48 hours	Group 1	47	0.4651	0.82661
	Group 2	47	0.5581	0.62877
VAS 72 hours	Group 1	47	0.1488	0.33479
	Group 2	47	0.2535	0.48371
NPRS 72 hours	Group 1	47	0.186	0.39375
	Group 2	47	0.2326	0.42746

**Table 2:** Intergroup comparison of difference in means of two groups using independent t-test p - level of significance; group 1 - rotary single file system; group 2 - reciprocating single file system; VAS - Visual Analogue Scale; NPRS - Numerical Pain Rating Scale *p≤0.05 was considered statistically significant

	t-test for equality of means					
	t	df	p-value	Mean difference	95% confidence interval of the difference	
					Lower	Upper
VAS 24 hours	.555	84	.581	.13256	-.34276	.60787
NPRS 24 hours	1.063	84	.291	.20930	-.18207	.60068
VAS 48 hours	-.977	83	.331	-.13915	-.42239	.14410
NPRS 48 hours	-.587	84	.559	-.09302	-.40798	.22193
VAS 72 hours	-1.167	84	.247	-.10465	-.28305	.07375
NPRS 72 hours	-.525	84	.601	-.04651	-.22276	.12974

**Table 3:** Comparison between each pair (say VAS 24 hours and VAS 48 hours) and (say NRPS 24 hours and NRPS 48 hours) using post-hoc test in group 1 p - level of significance; group 1 - rotary single file system; factor 1 - mean pain value; I-J - difference between pair of groups, 1, 2, and 3 in first and second columns indicate mean pain values at 24 hours, 48 hours, and 72 hours, respectively. *p≤0.05 was considered statistically significant.

(I) factor1	(J) factor1	Mean difference (I-J)		Std. error		p-value		95% confidence interval for difference^c^			
								Lower bound		Upper bound	
		VAS	NPRS	VAS	NPRS	VAS	NPRS	VAS	NPRS	VAS	NPRS
1	2	.772^*^	.605^*^	.134	.106	.000	.000	.438	.340	1.106	.869
	3	.967^*^	.884^*^	.166	.134	.000	.000	.553	.550	1.382	1.218
2	1	-.772^*^	-.605^*^	.134	.106	.000	.000	-1.106	-.869	-.438	-.340
	3	.195^*^	.279^*^	.069	.096	.022	.017	.022	.040	.368	.519
3	1	-.967^*^	-.884^*^	.166	.134	.000	.000	-1.382	-1.218	-.553	-.550
	2	-.195^*^	-.279^*^	.069	.096	.022	.017	-.368	-.519	-.022	-.040

**Table 4:** Comparison between each pair (say VAS 24 hours and VAS 48 hours) and (say NRPS 24 hours and NRPS 48 hours) using post-hoc test in group 2 p - level of significance; group 2 - reciprocating single file system; factor 1 - mean pain value, I-J - difference between pair of groups, 1, 2, and 3 in first and second columns indicate mean pain values at 24 hours, 48 hours, and 72 hours, respectively *p≤0.05 was considered statistically significant.

(I) factor1	(J) factor1	Mean difference (I-J)		Std. error		p-value		95% confidence interval for difference^c^			
								Lower bound		Upper bound	
		VAS	NPRS	VAS	NPRS	VAS	NPRS	VAS	NPRS	VAS	NPRS
1	2	.490^*^	.302^*^	.088	.078	.000	.001	.271	.107	.710	.498
	3	.714^*^	.628^*^	.131	.105	.000	.000	.388	.365	1.041	.891
2	1	-.490^*^	-.302^*^	.088	.078	.000	.001	-.710	-.498	-.271	-.107
	3	.224^*^	.326^*^	.068	.072	.006	.000	.054	.145	.393	.506
3	1	-.714^*^	-.628^*^	.131	.105	.000	.000	-1.041	-.891	-.388	-.365
	2	-.224^*^	-.326^*^	.068	.072	.006	.000	-.393	-.506	-.054	-.145

Study participants with no pain increased from 34% at 24 hours to 74% at 72 hours and 30% at 24 hours to 70% at 72 hours in the rotary and reciprocating groups, respectively. No patient had moderate or severe pain at 72 hours in both group 1 (Table 5) and group 2 (Table 6). No patient had a flare-up during any of the assessed periods in either group. One patient from both groups gave a history of drug intake at 24 hours. No statistically significant difference in pain outcome was found between genders in both group 1 (Table 7) and group 2 (Table 8).

**Table 5:** Frequency of observation of people with no pain. mild, moderate, severe pain at 24 hours, 48 hours, 72 hours measured using two pain scale in group 1 Group 1 - rotary file system; VAS - Visual Analogue Scale; NPRS - Numerical Pain Rating Scale

Time period	No pain		Mild pain		Moderate pain		Severe pain	
Sample size (N=47)	VAS	NPRS	VAS	NPRS	VAS	NPRS	VAS	NPRS
24 hours	16/47	16/47	30/47	29/47	1/47	2/47	0	0
48 hours	29/47	29/47	18/47	16/47	0	2/47	0	0
72 hours	35/47	35/47	12/47	12/47	0	0	0	0

**Table 6:** Frequency of observation of people with no pain. mild, moderate, severe pain at 24 hours, 48 hours, 72 hours measured using two pain scales in group 2 Group 2 - reciprocating file system; VAS - Visual Analogue Scale; NPRS - Numerical Pain Rating Scale

Time period	No pain		Mild pain		Moderate pain		Severe pain	
Sample size (N=47)	VAS	NPRS	VAS	NPRS	VAS	NPRS	VAS	NPRS
24 hours	14/47	14/47	31/47	32/47	2/47	1/47	0	0
48 hours	22/47	22/47	25/47	25/47	0	0	0	0
72 hours	33/47	33/47	14/47	14/47	0	0	0	0

**Table 7:** Mean pain values of males and females of group 1 (Rotary single file system) p - level of significance; VAS - Visual Analogue Scale; NPRS - Numerical Pain Rating Scale *p≤0.05 was considered statistically significant.

	Sex	N	Mean	Std. deviation	p-value	Inference
VAS 24 hours	Male	21	1.2526	1.33474	0.522	There is no significant difference
	Female	26	1.0083	1.14395		
VAS 48 hours	Male	21	.3421	.50806	0.902	There is no significant difference
	Female	26	.3458	.73779		
VAS 72 hours	Male	21	.1789	.33760	0.606	There is no significant difference
	Female	26	.1250	.33783		
NPRS 24 hours	Male	21	1.1053	.99413	0.804	There is no significant difference
	Female	26	1.0417	1.04170		
NPRS 48 hours	Male	21	.5263	.77233	0.671	There is no significant difference
	Female	26	.4167	.88055		
NPRS 72 hours	Male	21	.2632	.45241	0.258	There is no significant difference
	Female	26	.1250	.33783		

**Table 8:** Mean pain values of males and females of group 2 (reciprocating single file system) p - level of significance; VAS - Visual Analogue Scale; NPRS - Numerical Pain Rating Scale *p≤0.05 was considered statistically significant.

	Sex	N	Mean	Std. deviation	p-value	Inference
VAS 24 hours	Male	19	.7824	.84056	0.281	There is no significant difference
	Female	28	1.1154	1.05705		
VAS 48 hours	Male	19	.4062	.49996	0.561	There is no significant difference
	Female	28	.5308	.76669		
VAS 72 hours	Male	19	.2235	.50932	0.747	There is no significant difference
	Female	28	.2731	.47544		
NPRS 24 hours	Male	19	.6471	.70189	0.162	There is no significant difference
	Female	28	1.0000	.84853		
NPRS 48 hours	Male	19	.5294	.51450	0.812	There is no significant difference
	Female	28	.5769	.70274		
NPRS 72 hours	Male	19	.1765	.39295	0.493	There is no significant difference

## Discussion

This study attempted to standardize the most known confounding factors of pain from previous studies, including preoperative pain, pulpal and periapical status, patient demographic factors, tooth type and root canal anatomy, drug intake history, and operator and treatment procedures. Since mechanical allodynia is a significant postoperative root canal treatment pain determinant, this study included only patients without preoperative pain. This could also be the reason for the study's low mean postoperative pain outcome in both groups. The results of studies comparing the apical debris extrusion using different single-file systems with two different motion kinetics have been contradictory. Some in-vitro studies suggest more debris extrusion associated with rotary than reciprocating file systems [9,10], while other studies have proved the opposite [11-13]. On the contrary, the two studies did not significantly differ in apical debris extrusion between the two groups [14,15]. Reduction in the number of files and the balanced force concept used in the reciprocating motion have been suggested as the major reasons for this group's reduced apical debris extrusion. Studies favoring rotary motion have attributed it to the screw conveyor effect, which pulls the collected debris coronally. An in vitro study similar to this current study using the same file systems was done by Guls et al. (2018), and the study could not find a significant difference between the two groups [15]. Hence, the suggested reasons must be evaluated in the absence of numerous other confounding factors present in these previous studies. This study also attempted to nullify the effect of foraminal enlargement of necrotic teeth on apical debris extrusion by using an instrument that caused similar foraminal enlargement of 0.25mm in both groups. Considering the pioneering work of Torabinejad et al., stating the influence of age on postoperative pain, participants between 20-40 years were selected [16]. The current study did not find any difference in the occurrence and intensity of pain among males and females, similar to the findings of previous studies [17,18]. Non-vital teeth having a lesion of less than 3mm were included to reduce any confounding bias if at all present [19]. Since canal debridement can differ in root canal complexities, the present study included only teeth with a single straight root canal. This current study excluded patients with a history of drug intake within one week before treatment. Since operator skill and efficiency affect treatment and outcome, the study procedure was carried out by a single operator blinded to the pain outcome. The creation of a glide path with reciprocating or rotating instruments caused a similar degree of postoperative pain, according to Keskin et al. [20].

The VAS and the NPRS scales were used because they have greater sensitivity to change [21,22]. Hence greater credibility for obtained results could be established, unlike many previous studies, where only one pain scale was assessed. Pain after biomechanical preparation was evaluated, unlike previous studies where the pain was assessed after a single visit root canal treatment [23]. Hence, this study had no confounding factors related to root canal obturation. The results of our study are in accordance with the clinical trials of Yilmaz et al., Çanakçi et al., and Mollasahi et al., stating no difference between reciprocating and continuous rotation groups [24-26]. The study done by Oliveira et al. was similar to the present study in that it assessed pain after biomechanical preparation in teeth with chronic apical periodontitis using the VAS and NPRS pain scale. However, the study differed in the file system (Protaper Next and Reciproc file) used, tooth analysis (maxillary and mandibular molars), and the instrumentation protocol followed. The study found that the frequencies of postoperative pain were similar between groups [18]. The enhanced physical properties and improved instrument design could be the reason for low debris extrusion and low pain outcome in both groups, as found by Guls et al. [15].

Limitations of the study

One of the main concerns about studying pain is the subjectiveness of the evaluation. Each person's threshold for pain is unique, and despite all the precautions taken, it is not always possible to control all potential sources of pain that may lead to bias. In addition, because only single-rooted teeth with one canal were investigated in this study, caution is needed before generalizing the results to multi-rooted teeth. Even though the apical foraminal enlargement and apical taper of a few millimeters could be standardized, the two file systems differed in their cross-section, flute design, and number, alloy composition, metallurgical properties, etc. These may act as confounding factors in the outcome. The study assessed pain after biomechanical preparation, the outcome of the study in the single-visit treatment is yet to be determined.

## Conclusions

Within the limitations of the study, it can be concluded that postoperative pain was of low intensity for patients who received root canal treatment with either the Hyflex EDM /rotary single file group or Reciproc blue/reciprocating single file group at all the time points assessed. The patients' intake of medication was similar regardless of the instrumentation technique. The mean pain value was found to be maximum at 24 hours and least at 72 hours in both rotary and reciprocating file groups. The difference in pain outcome was statistically significant between 24 hours, 48 hours, and 72 hours (p<0.05). Hence, this study concluded that the instrumentation kinematics (single-file reciprocating or single-file rotary) had no impact on the intensity of postoperative pain after biomechanical preparation of root canals and no file system is superior to the other in terms of postoperative pain and both file systems can be used clinically with equal efficiency when considering postoperative patient discomfort.

## Figures and Tables

**Figure 8:**
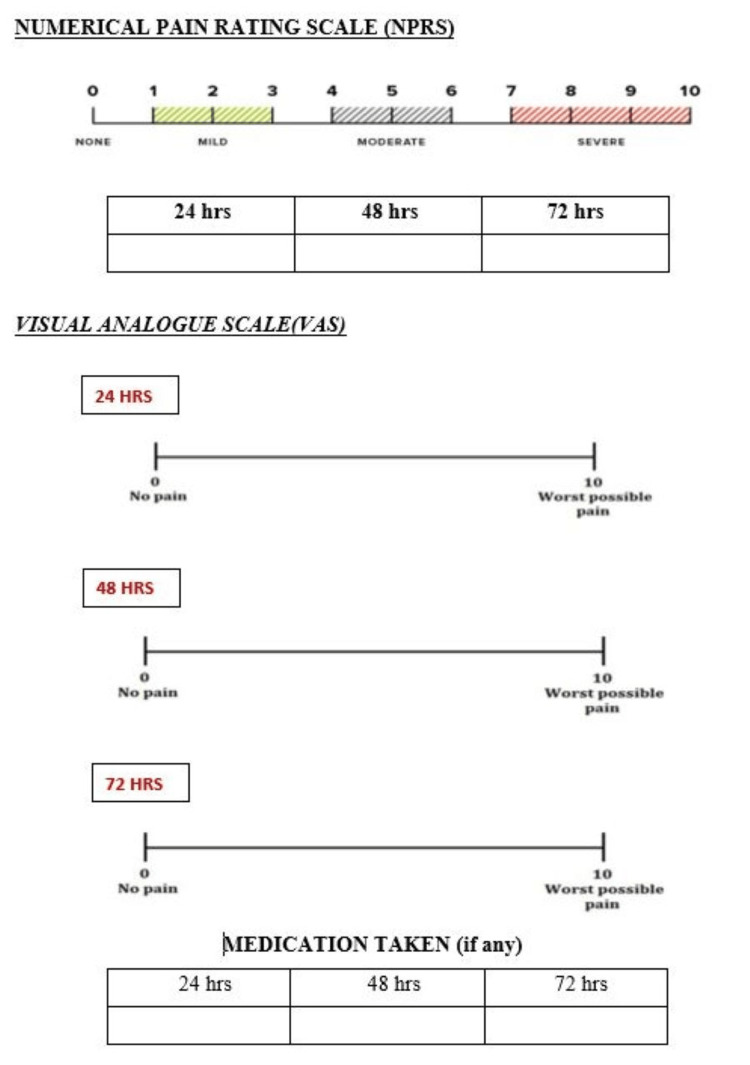
Pain questionnaire

## Appendices

Chief complaint

History of presenting illness

Medical history

Diabetes                                           Pregnancy

Hypertension                                    lactation

asthma                                              Gastrointestinal/renal

cardiac disease                                 Hematologic

Neurologic                                       Other diseases

Medications

Drug Allergy

Past Dental History

General Examination

BP                                       Pulse

Weight                                Height

Extra Oral Examination

Intra Oral Examination

Soft tissue

Hard tissue

Caries

Clinical tests

Pulp sensibility tests

                  Cold test 

                  Electric Pulp Test

Periradicular tests

                   Percussion

                   Palpation

Radiographic Examination

Diagnosis

Working length (recorded by both electronic apex locator and radiographic method):

Instrument used for BMP as randomly selected-

Post-operative pain assessment done using VAS&NPRS (Table 9)

**Table 9:** Post-operative pain assessment done using VAS &NPRS

	No pain	Mild pain	Moderate pain	Severe pain
24 hrs				
NPRS				
VAS				
48 hrs				
NPRS				
VAS				
72 hrs				
NPRS				
VAS				

**Table 10:** Analgesic usage (if any, as obtained from patient after three days)

24 hrs	48 hrs	72 hrs
